# The Protective Role of Melatonin in Sperm Cryopreservation of Farm Animals and Human: Lessons for Male Fish Cryopreservation

**DOI:** 10.3390/ani12060791

**Published:** 2022-03-21

**Authors:** Alexandra I. Alevra, Athanasios Exadactylos, Eleni Mente, Serafeim Papadopoulos

**Affiliations:** 1 Hydrobiology-Ichthyology Laboratory, Department of Ichthyology and Aquatic Environment, University of Thessaly, Fytokou Str., 38446 Volos, Greece; exadact@uth.gr; 2 Laboratory of Ichthyology-Culture and Pathology of Aquatic Animals, School of Veterinary Medicine, Aristotle University of Thessaloniki, University Campus, 54006 Thessaloniki, Greece; emente@uth.gr

**Keywords:** cryopreservation, fish, farm animals, human, semen, melatonin, antioxidant, oxidative stress

## Abstract

**Simple Summary:**

In recent years, cryopreservation of fish sperm has been a rapidly evolving technique that contributes both to the improvement of genetic reproduction programs and the proper management of broodstock as well as to ensuring the viability of endangered species. However, this technique can cause significant damage to sperm, making the use of cryoprotectants and antioxidants in cryopreservation solutions imperative. The hormone melatonin has demonstrated positive effects on the cryopreservation of sperm in both farm animals and humans. Therefore, the plethora of research that has been conducted on animals and humans could be expanded to fish cryopreservation, making melatonin potentially a very promising alternative cryoprotectant.

**Abstract:**

Cryopreservation is a technique that offers various advantages, especially in fish, among others, that makes the reproduction of species easier through a constant supply of sperm, synchronization of the gamete availability of both sexes, storage of semen for genetic improvement programs, reduction in the cost by eliminating the need to maintain male broodstock, and conserving the gametes of endangered species. However, freezing and warming procedures for cryopreservation lead to a reduction in the quality and viability of cryopreserved sperm because of oxidative stress. For this reason, the enrichment of extender media with antioxidants is a common method of cryopreservation of the semen of several fish species. Recently, many studies have been published for the protective role of antioxidants and especially of melatonin on male fertility preservation both in farm animals and humans, demonstrating the beneficial effects of melatonin as a sperm cryoprotectant. On the other hand, very few studies were conducted using melatonin as an antioxidant in different male fish species for semen cryopreservation. We conclude that the use of moderate concentrations of melatonin are beneficial to semen preservation, and the mechanisms through which melatonin acts positively on spermatozoa need to be further investigated to establish improvement protocols for cryopreservation in fish species.

## 1. Introduction

The use of the technology of sperm cryopreservation offers many benefits extensively described in previous reviews for fish [1,2,3], farm animals [4,5,6], and humans [7,8]. In addition, extensive reviews have been published that include detailed protocols for fish species [9,10,11,12].

The development of fish sperm cryopreservation protocols for marine species is not as extensive for freshwater species, and the plethora of research work concerns the latter [2]. The main goal of most work on new species is to maintain stocks to ensure production, to optimize genetic improvement programs, and to properly manage offspring [2,13]; therefore, the development of cryopreservation protocols would help to achieve the above objectives [2]. In addition, cryopreservation of gametes can be used to protect endangered species [14].

The main goal of this brief review was to summarize the findings in the literature that refers to the supplementation of melatonin in cryopreservation media of semen, both in humans and farm animals, and to provide this knowledge in the cryopreservation of fish milt.

## 2. Cryodamage of Spermatozoa

The cryopreservation of sperm provokes a decrease in its quality and viability, mainly due to the increase in the production of reactive oxygen species (ROS) and the alteration of oxidative metabolism during the process of freezing and warming [15].

Although the sperm of fish, like all biological systems, are provided with protective antioxidants agents [16,17], in the cryopreservation technique, the antioxidant defense of the sperm is almost insufficient due to the reduced amount of these factors after the dilution of sperm [18,19]. As a consequence, during cryopreservation, an imbalance is observed between ROS production and the inherent antioxidant system [20,21], known as oxidative stress. Scientific studies in fish have shown that ROS production during cryopreservation contributes to the occurrence of lesions in sperm [22,23], resulting in lipid peroxidation (LPO) [24,25], DNA fragmentation [18,26], mitochondrial damage and dysfunction [23,27,28,29], protein oxidation [30], and loss or inactivation of enzymes associated with sperm motility [24,31,32]. Due to the aforementioned problems, it has become common practice to enrich the cryopreservation diluents of the sperm of many fish species with enzymatic and non-enzymatic antioxidants [18,33,34,35]; nevertheless, their use is often controversial.

## 3. Antioxidant Supplementation of Semen Extenders: The Case of Melatonin

Considering that the increased production of ROS during the cryopreservation process is partly responsible for the poor quality of sperm after thawing, various antioxidants have been proposed and tested for the cryopreservation of sperm of various terrestrial animals and fish [36]. Recent published studies focus on the protective role of various antioxidants, especially melatonin, in maintaining male fertility in both productive animals [37,38] and fish species [39], thus demonstrating the increased interest in this hormone.

Melatonin, the principal hormone secreted by the pineal gland, has been suggested as a free radical scavenger and antioxidant [40]. Mainly due to the fact of its amphiphilic nature that allows it to pass through all morphophysiological barriers of the cell; it is one of the most effective antioxidants protecting cells from oxidative stress caused by reactive species [41]. In addition, its lipophilic nature allows it to easily cross cell membranes and act directly in various organs including those of the reproductive system [42,43]. Of particular interest is the fact that melatonin’s metabolites, which are formed when the hormone functions as a scavenger, are likewise equally as good or better than the parent molecule in neutralizing toxic oxygen-based and nitrogen-based reactants [44].

The cytoprotective action of melatonin and its metabolites is due to the fact of its direct and indirect antioxidant properties [45]. The direct properties include the scavenging of both ROS and RNS (reactive nitrogen species) [46], while the indirect effects cover the stimulation of antioxidative enzymes and inhibition of pro-oxidative enzymes [47], probably through epigenetic mechanisms [48]. This molecule with its strong detoxifying effect at the mitochondrial level, could be an appropriate candidate for improving the quality of animal sperm during cryopreservation [49,50]. It has been observed that this substance could protect sperm from oxidative damage [51], maintain its viability [42,52], and reduce morphological abnormalities [53,54] and DNA fragmentation [55]. Improvement in sperm quality due to the high levels of endogenous melatonin has been found in humans [56], while in vitro treatment with this hormone can improve human sperm motility [56] and several quality parameters of ram [57] and pig [58] sperm. Finally, melatonin has been used as an additive antioxidant in the cryopreservation of sperm [59,60] helping to increase its quality after thawing [61].

## 4. Melatonin Supplementation in Farm Animals and Human Freezing Medium

The majority of recent studies evaluating the effect of melatonin on the cryopreservation of sperm focus on cattle compared to those on sheep, pigs, or goats [38].

**Figure 1:**
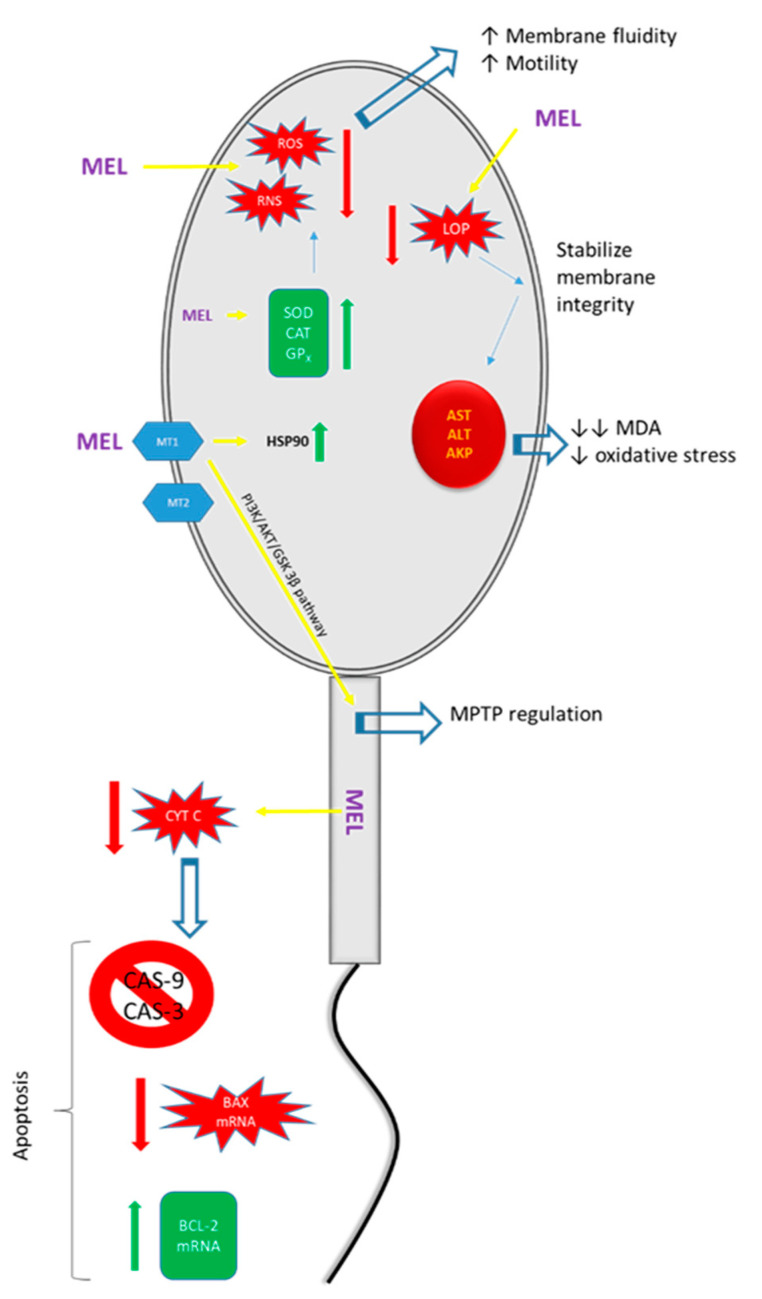
Illustration of the positive effects of melatonin (MEL) on spermatozoa. MT1: melatonin type 1 receptor; MT2: melatonin type 2 receptor; ROS: reactive oxygen species; RNS: reactive nitrogen species; LOP: lipid peroxidation; SOD: superoxide dismutase; CAT: catalase; GP_X_: glutathione peroxidase; HSP90: heat shock protein 90; AST: aspartate amino transferase; ALT: alanine aminotransferase; AKP: alkaline phosphatase; MDA: malondialdehyde; MPTP: mitochondrial permeability transition pore.

In summary, the effect of melatonin on sperm is shown in Table 1 and the pathway of action in Figure 1.

Several studies have shown that the cryoprotective effects of melatonin depend on its concentration [59,75]. In the international literature, many different concentrations have been defined as being optimal for the cryoprotection of sperm. Among others, some studies have shown that 1 and 2 mM are the optimal concentrations, while another study found that the best concentration was 0.25 mg/mL in various species [76]. In humans, a better range of sperm viability and motility were observed at a 3 mM melatonin concentration, and intracellular ROS levels were reduced [51]. The vitality of thawed human sperm was found to be improved after the supplementation of 0.1 mM melatonin, while it was adversely affected by other concentrations (i.e., 0.001 and 1 mM) [62]. Karimfar et al. [75] reported that the best protection for human sperm against cryopreservation damage was observed at a 0.01 mM melatonin concentration.

Studies have shown that parameters, such as membrane integrity, motility and velocity, capacitation, antioxidant protein quantity, and developmental competence of fresh and frozen sperm improved after administration of moderate melatonin concentrations [62,77,78,79]. The addition of melatonin to cryopreservation solutions of bovine sperm [71], sheep [80,81], goats [82,83], rams [70], buffalo [84], and pigs [85,86] increased the number of live sperm with normal quality after thawing, including the normal length and movement of the tail, and reduced morphological defects of the sperm. In addition, in farmed animals, the frozen sperm with membrane integrity showed greater motility [60,86].

The scientific work on the role of melatonin in the mitigation of oxidative damage, mostly concerns humans and fewer farm animals. Significant progress has been made in understanding the action of melatonin against oxidative damage caused by cryopreservation [38]. In studies of farm animals, the available data focus on the positive effects of melatonin on sperm quality indicators but without clearly identifying the mechanisms by which it acts. It would therefore be crucial to further investigate these mechanisms in both fresh and frozen sperm, especially in sheep, goats, and pigs [38].

## 5. Studies Used Melatonin as an Antioxidant in Fish Sperm Cryopreservation

Very few studies have been conducted using melatonin as an antioxidant in different male fish species for semen cryopreservation compared to farm animal species.

In freshwater species, curimba (*Prochilodus lineatus*), melatonin was used as an additive in the sperm cryopreservation extender and no significant differences were found among different concentrations (i.e., 1, 2, and 3 mM), neither in motility parameters and spermatozoa morphological anomalies nor in fertilization capacity [87].

Recently, in the same fish, *Prochilodus lineatus*, Motta et al. [88] studied the antioxidant capacity of four different concentrations of melatonin (i.e., 2.00, 2.75, 3.50, and 4.25 mM) by determining the kinetic motility parameters and oxidative stress indices in sperm after thawing. The use of high concentrations of melatonin was shown to be harmful to the sperm of this species. In addition, 2.00 mM melatonin resulted in a higher curvilinear velocity (VCL) and linear velocity (VSL) and reduced catalysis (CAT). However, with the use of this dosage, no statistically significant differences were observed with the control in motility rate, membrane integrity, percentage of normal cells, lipid peroxidation, and fertilization and hatching rates.

Ferrão [89] examined the protective action of melatonin during sperm cryopreservation in F1 *Senegalese sole* using two different melatonin supplementations (i.e., 0.1 and 10 mM). In the post-thawed sperm, motility, viability, DNA fragmentation, lipid peroxidation, ROS, and apoptosis were examined. The 10 mM melatonin supplement exhibited significantly lower spermatozoa viability combined with higher percentages of late apoptotic cells dead by caspases. The post-thawed sperm motility decreased throughout post-activation time in the control and in the melatonin supplemented groups (i.e., 0.1 and 10 mM).

In Portugal, Félix et al. [90], for first time, evaluated the antioxidant capacity of three concentrations of melatonin (i.e., 0.001, 0.01, and 0.1 mM) during sperm cryopreservation of gilthead seabream (*Sparus aurata*). The motility of all parameters, which analyzed total motility (TM), progressive motility (PM), curvilinear velocity (VCL), straight line velocity (VSL), and linearity (LIN), were revealed to be influenced by melatonin. Regarding DNA fragmentation, no differences were observed between treatments in tail DNA (%), but olive tail movement was consistently higher at melatonin treatment. Moreover, they brought into account another parameter of the action of melatonin during cryopreservation. The effect of endogenously produced melatonin by night on the spermatozoa quality. The authors compared sperm samples that were collected at two different chronic points, mid-light and mid-dark. Interestingly, they concluded that the higher levels of endogenous melatonin production at night could have an important role in spermatozoa protection during cryopreservation [91].

Finally, Palhares et al. [92] verified the percentage of dead and live spermatozoa of frozen *Brycon orbignyanus* semen after freezing with extenders containing different concentrations of melatonin (i.e., 1 and 2 mM) and after three different dry shipper freezing times (i.e., 15 min, 12 h, and 24 h). The authors concluded that the addition of 2 mM of melatonin in freezing medium with the cryoprotectant methylglycol improved *Brycon* *orbignyanus* sperm quality regarding sperm kinetics, while the tested freezing times did not influence the quality of thawed semen. Additionally, the same group in Brazil [93], studied melatonin supplementation in different freezing curves determining the antioxidant enzyme activity and the peroxidation lipid and sperm characteristics of cryopreserved *Brycon orbignyanus* milt. Melatonin concentrations of 1 and 2 mM were examined, and significant differences in viability, morphology, motility, and fertilization rate in the solutions with melatonin were identified. Specifically, total motility, progressive motility, and motility time were significantly different. In terms of oxidative stress markers, the solutions with melatonin yielded the lowest values. The study concluded that 2 mM of melatonin is beneficial for *Brycon orbignyanus* semen based on the best sperm characteristics, peroxidation lipid, and antioxidant enzyme activity obtained during cryopreservation.

## 6. Conclusions: Lessons to Be Learned from Farm Animals and Human Studies

In the future, research should be carried out on the improvement of mechanisms that trigger the production of endogenous antioxidants, such as melatonin, to protect spermatozoa naturally from oxidative stress, as other research groups support. Definitely, the need for a description of appropriate and safe application methods in regard to male fertility cryopreservation in different fish species is required as soon as possible.

The findings of previous research papers on farm animals and humans are expected to be helpful for the improvement in cryopreservation protocols in fish species. The success of freezing protocols could be of help in strengthening the programs of intensive breeding and genetic improvement in aquaculture industry [94]. In particular, this will be achieved through transmission and application of acquired knowledge of farm animals and human semen cryopreservation to ensure the reproductive efficiency and productivity of fishes.

All of this knowledge could lead to the following conclusions:

1. Not all melatonin concentrations are optimal for the properties of sperm cryopreservation extenders;
2. Improvements in the use of melatonin as an antioxidant to moderate oxidative damage is more advanced in humans than in farm animals due to the spermatozoon’s smaller head which presents maximum cryostability [95];
3. Use of moderate concentrations of melatonin improves the quality of both fresh and frozen semen;
4. The positive effects of melatonin on spermatozoa have been proved in non-seasonal long-day and short-day breeders, which suggests that this action is not relevant by the hypothalamus–pituitary–testicular axis regulation;
5. Because of its low toxicity and commonly accepted antioxidant activity, melatonin could be a perfect candidate to enhance semen quality during cryopreservation;
6. The mechanisms through which melatonin acts positively on spermatozoa need further investigation.

The findings of the research on the use of melatonin as an antioxidant/cryoprotectant in the cryopreservation media support the idea that melatonin concentration may be dependent on the species and should be tested for different fish species.

In a nutshell, our efforts are to establish protocols with detailed descriptions that are generally accepted by the research community in the field of cryopreservation of marine species sperm. The role and contribution of melatonin in these protocols as an antioxidant/cryoprotectant remain to be explored with the important help of researchers in the topics of genetic, cryobiology, and physiology of reproduction.

## Figures and Tables

**Table 1:** Effects of melatonin on sperm.

Pathway of Action	Εffects on Spermatozoa	References
Reduction in excessive production of free radicals.	Positive effects on the function and morphometry parameters of sperm in humans and various farm animals.	[62,63,64]
Upregulation of the expression of heat shock protein (HSP) 90.	Resistance to stress factors in frozen–thawed sperm.	[62]
Upregulation of antioxidant enzymes, e.g., superoxide dismutase, glutathione peroxidase, and catalase.	Elimination of ROS levels causing preservation of membrane fluidity and motility.	[62,65,66]
Regulation of mitochondrial permeability transition pores (MPTPs) as a result of binding to the MT1 receptor and the activation of the PI3K/AKT/GSK 3β pathway.	Improvement in the quality and fertilizing capacity of frozen–thawed ram sperm.	[67]
Reduction of LPO production leads to (a) stabilization of membrane integrity and (b) prevention of leakage of intracellular enzymes, e.g., aspartate transaminase (AST), alanine transaminase (ALT), and phosphatase.	Decreased malondialdehyde (MDA) concentrations and oxidative stress.	[68,69]
Enhancement of the functions of antioxidant enzymes.	Protection against oxidative modifications of DNA. DNA becomes more resistant to fragmentation, reducing the rate of sperm degradation and enhancing its viability and functions.	[42,59,70,71]
Action as an anti-apoptotic molecule.	(a) Reduction of mitochondrial CYT C release and inhibition of the activation of CAS-9 and CAS-3 proteins.	[72]
	(b) Reduction of pro-apoptotic BAX mRNA transcripts and increase in anti-apoptotic BCL-2 mRNA transcripts in bull and buck sperm.	[73,74]

## Data Availability

Not applicable.
